# Mediterranean Diet Adherence and Sleep Quality Among Primary and Secondary School Teachers in Portugal: An Exploratory Cross-Sectional Study

**DOI:** 10.3390/nu17182948

**Published:** 2025-09-12

**Authors:** Leandro Oliveira, António Raposo, Thamer Alslamah, Hani A. Alfheeaid, Nada Alqarawi, Marta Esgalhado

**Affiliations:** 1 CBIOS (Research Center for Biosciences and Health Technologies), ECTS (School of Health Sciences and Technologies), Lusófona University, Campo Grande 376, 1749-024 Lisboa, Portugal; marta.esgalhado@ulusofona.pt; 2 Department of Public Health, College of Applied Medical Sciences, Qassim University, Buraydah 51452, Saudi Arabia; 4037@qu.edu.sa; 3 Department of Food Science and Human Nutrition, College of Agriculture and Food, Qassim University, Buraydah 51452, Saudi Arabia; h.alfheeaid@qu.edu.sa; 4 Department of Psychiatric and Mental Health, and Community Health, College of Nursing, Qassim University, Buraydah 51452, Saudi Arabia; n.alqarawi@qu.edu.sa

**Keywords:** Mediterranean diet, sleep quality, teachers, cross-sectional study, Portugal

## Abstract

Background: Diet and sleep are modifiable lifestyle factors that influence health and well-being. While adherence to the Mediterranean diet has been associated with improved sleep quality, this relationship remains understudied in high-stress occupational groups such as school teachers. Objective: To examine the association between adherence to the Mediterranean diet and self-reported sleep quality among primary and secondary school teachers in Portugal. Methods: A cross-sectional study was conducted with 113 teachers who completed a self-administered digital questionnaire. Adherence to the Mediterranean diet was assessed using the 14-item Mediterranean Diet Adherence Screener (MEDAS) and sleep quality was evaluated with the Pittsburgh Sleep Quality Index (PSQI). Results: Of the 113 participants, 58% reported poor sleep quality (PSQI > 5), and the median PSQI score was 6.0 (IQR 4.0–8.8). The median MEDAS score was 9.0 (IQR 8.0–10.0), with 34% classified as having high adherence. When stratified by dietary adherence, there were no statistically significant differences in global PSQI or its components, and multivariable linear regression showed no association between MEDAS and PSQI. Patterns varied by teaching level: poor sleep predominated in most levels except the 2nd Cycle; high adherence was most prevalent in the 1st Cycle, moderate adherence predominated in the 3rd Cycle and secondary education, and the 2nd Cycle showed similar shares of moderate and high adherence. Conclusions: In this occupational group, poor sleep was common and varied across teaching levels, while Mediterranean diet adherence showed no detectable association with overall sleep quality in this study. Larger, longitudinal studies using objective assessments of diet and sleep, and accounting for factors such as stress and chronotype, are warranted to clarify potential links in educational settings.

## 1. Introduction

The Mediterranean diet is internationally recognised as both a cultural and nutritional model of excellence [1]. Originating from the traditional dietary practices of populations in southern Europe, particularly Greece, Italy, and Spain, it is characterised by a high consumption of fruits, vegetables, whole grains, legumes, nuts, and olive oil, moderate intake of fish and dairy products, low consumption of red and processed meats, and moderate wine consumption with meals [2]. Numerous studies have demonstrated its protective effects against cardiovascular disease, type 2 diabetes, certain types of cancer, and neurodegenerative conditions [3,4,5]. Furthermore, evidence suggests that adherence to the Mediterranean diet may positively influence psychological well-being and sleep health, likely via mechanisms related to inflammation, nutrient density, and circadian regulation [6]. Portugal, as part of the Mediterranean region, shares many of these dietary principles. However, recent data indicate moderate to low adherence among the Portuguese population. A national survey during the COVID-19 pandemic found that high adherence was limited to approximately 26% of respondents [7]. Segmentation studies in Portugal have identified groups such as “olive-oil lovers” but also highlighted barriers to adherence including cultural norms, economic constraints, and difficulty integrating dietary patterns into daily life [8]. Moreover, traditional eating patterns in Portugal also encompass the Atlantic diet, particularly in the north, which emphasises seafood, dairy, bread, and red meats [9]. These regional and cultural influences help explain the variability in adherence to the Mediterranean diet across the country.

Sleep is a vital physiological function essential for cognitive performance, emotional balance, metabolic regulation, and overall quality of life [10,11,12]. However, modern lifestyles and work-related stress have contributed to a global increase in sleep-related problems [13,14]. Poor sleep quality and insufficient duration have been linked to a range of adverse health outcomes, including obesity, hypertension, insulin resistance, mood disorders, and impaired immune function [15,16]. Given its crucial role in health and daily functioning, sleep has become a growing area of concern in public health research [17]. Identifying modifiable factors that promote better sleep quality is therefore of significant importance [18].

Among these factors, diet has emerged as a key element influencing sleep. Nutrients commonly present in the Mediterranean diet, such as tryptophan, magnesium, omega-3 fatty acids, B vitamins, and polyphenols, are known to play roles in the synthesis and regulation of serotonin and melatonin, which are involved in the sleep–wake cycle [19,20,21]. Additionally, the diet’s anti-inflammatory and antioxidant properties may reduce systemic inflammation and oxidative stress, both of which are associated with sleep disturbances [22]. Observational studies suggest that individuals who adhere more closely to the Mediterranean diet tend to experience better sleep quality, longer sleep duration, and fewer insomnia symptoms [23].

Despite these promising associations, the available evidence remains limited in scope. Most studies have been conducted in general population samples or among older adults, while relatively few have explored this relationship in working-age individuals or in specific professional groups. One group that merits particular attention is school teachers, who often experience high levels of occupational stress and demanding workloads [24]. Teaching is an emotionally and cognitively taxing profession that involves long working hours, administrative responsibilities, and the ongoing challenge of meeting diverse student needs [25]. These stressors are frequently associated with fatigue, burnout, and sleep disturbances [26,27].

In addition to sleep difficulties, stress may negatively affect dietary habits among teachers. Time constraints, emotional exhaustion, and lack of work–life balance can lead to irregular eating patterns, increased consumption of processed foods, and reduced intake of fresh, nutrient-dense meals [28]. These behaviours may reduce adherence to the principles of the Mediterranean diet and contribute to a cycle in which poor sleep and poor diet reinforce one another [29,30]. Evidence suggests that inadequate sleep can increase cravings for high-calorie foods and reduce the motivation to engage in healthy behaviours [31,32]. In turn, a low-quality diet may negatively affect sleep by disrupting hormonal regulation and increasing inflammation [33,34].

Although the interaction between diet and sleep is increasingly recognised in the scientific literature, research specifically targeting teachers remains scarce. This is a critical gap, as teachers not only face particular health vulnerabilities due to their work environment but also serve as influential role models for children and adolescents [35]. Promoting healthy behaviours among teachers may therefore have benefits that extend beyond their individual health, potentially contributing to more supportive and health-promoting school environments [36,37].

Furthermore, most existing studies on the Mediterranean diet and sleep have been conducted in countries where adherence to this dietary pattern is traditionally high [23]. There is a need for research in more diverse settings and among populations where dietary habits may differ, including professional groups whose working conditions influence both diet and sleep [38]. Teachers represent a particularly relevant group for this type of research, as they are often subject to occupational challenges that compromise their ability to maintain healthy routines [39].

Understanding whether adherence to the Mediterranean diet is associated with better sleep quality in this group could inform the development of targeted health promotion interventions [40]. Such interventions might be implemented within schools and integrated into broader strategies to support teacher well-being [41]. By focusing on both dietary and sleep-related behaviours, these programmes could contribute to improved physical and psychological health, reduced absenteeism, and enhanced professional performance [38]. Globally, insufficient sleep is highly prevalent and strongly linked to multiple chronic diseases, representing a major public health priority [14,42]. In Portugal, population-based data indicate a considerable burden of sleep complaints, including frequent insomnia symptoms [43]. In parallel, adherence to the Mediterranean diet has shown erosion in Southern Europe and national monitoring in Portugal underscores the need for nutritional promotion strategies [44,45,46]. In the educational sector, occupational stress among teachers is consistently reported as high, potentially affecting both dietary habits and sleep quality, which justifies integrated workplace health promotion approaches [24,47].

From a clinical practice standpoint, food literacy at first contact with specialized care, such as diabetology services, is critical to tailor counselling and to address early gaps in nutrition knowledge [48]. Likewise, across the life course, nutrition plays a central role in healthy aging and in the reduction of age-related chronic disease risk [49]. Considering the bidirectional links between diet quality and sleep, jointly screening these behaviours may help clinicians identify at-risk individuals who could benefit from integrated lifestyle support [50,51]. These considerations motivated us to examine whether Mediterranean diet adherence relates to sleep quality in a high-stress occupational group.

The aim of this exploratory study is to examine the association between adherence to the Mediterranean diet and self-reported sleep quality among primary and secondary school teachers. Specifically, the study seeks to determine whether higher adherence to this dietary pattern is associated with better sleep outcomes and to identify sociodemographic and lifestyle factors that may influence this relationship within the teaching profession. Understanding these associations is not only relevant from an epidemiological standpoint but also has practical implications for clinical and occupational health. Teachers face unique challenges, including high stress levels and irregular work schedules, which may compromise both dietary habits and sleep. Identifying modifiable factors such as diet and sleep hygiene could inform targeted interventions to improve health outcomes and work performance in this professional group.

## 2. Materials and Methods

### 2.1. Procedure

This descriptive cross-sectional study, which received ethical clearance from the Ethics Committee of the School of Health Sciences and Technologies at Universidade Lusófona (approval code P04-23) on 31 March 2023, was reported in accordance with the Strengthening the Reporting of Observational Studies in Epidemiology (STROBE) guidelines, as recommended by the EQUATOR Network, to ensure transparent and complete reporting. A completed STROBE checklist is provided as Supplementary Materials (Table S1). Eligible participants were active primary and secondary school teachers, aged 18 or above, working in public or private educational institutions in Portugal. Exclusion criteria included not currently working as a primary or secondary school teacher at the time of data collection, inability to understand or respond to the questionnaire in Portuguese, and self-reported medical diagnosis or condition that could significantly affect sleep patterns or dietary habits (e.g., severe sleep disorders, neurodegenerative diseases, or specific therapeutic diets).

Data were collected during April and December 2023 using a self-administered digital questionnaire. The estimated completion time was approximately 15 min. The survey was distributed online through institutional mailing lists, personal networks, and various digital platforms, including WhatsApp^®^ (https://www.whatsapp.com/), Facebook^®^ (https://www.facebook.com/), and Instagram^®^ (https://www.instagram.com/). The outreach was supported by collaborations with schools and professional associations with whom the research team maintained established academic or professional links.

To ensure that only eligible individuals participated, the questionnaire included an initial confirmation item requiring respondents to declare their professional role as a school teacher. Further questions about school type, teaching level, and years of experience were included to verify consistency in responses. All participants provided informed digital consent prior to accessing the questionnaire. Participation was entirely voluntary and respondents were free to exit the survey at any moment by simply closing the browser. Data collection was anonymous and all information was securely stored on an encrypted institutional server.

### 2.2. Instruments

The survey instrument, written in Portuguese, consisted of three relevant sections: personal and professional background, dietary intake, and sleep quality.

The first section gathered sociodemographic data such as sex, age, and geographical region (classified according to NUTS II) and educational attainment, along with job-related variables including teaching level (1st, 2nd, or 3rd Cycle of primary education, or secondary education) and years of professional experience. Lifestyle information was also recorded, including tobacco use and self-reported weight and height. Body Mass Index (BMI) was calculated using the standard formula (weight in kilograms divided by height in meters squared) and nutritional status was categorised based on World Health Organization guidelines [52]. Physical activity levels were assessed using the International Physical Activity Questionnaire. To assess dietary behaviour, self-reported adherence to the Mediterranean diet was evaluated using the 14-item Mediterranean Diet Adherence Screener (MEDAS) [53]. This validated instrument includes 14 dichotomous items, each scored as 0 or 1, yielding a total score ranging from 0 to 14. Higher scores indicate greater alignment with the Mediterranean dietary pattern. In this study, the Portuguese validated version of the MEDAS was used [54]. For analytical purposes, adherence levels were categorised as low (≤5 points), moderate (6 to 9 points), and high (≥10 points), based on established thresholds [55].

Sleep quality was measured using the Pittsburgh Sleep Quality Index (PSQI), a validated and widely applied tool that examines self-reported sleep characteristics over the preceding month [56]. The PSQI consists of 19 items grouped into seven domains: subjective sleep quality, sleep latency, sleep duration, sleep efficiency, sleep disturbances, use of sleep medication, and daytime dysfunction. Each domain is rated on a scale from 0 to 3, yielding a global score ranging from 0 to 21, with higher scores indicating poorer sleep quality. A global PSQI score above 5 suggests clinically relevant sleep impairment [56]. In this study, the Portuguese validated version of the PSQI was used [57].

### 2.3. Statistical Analysis

Descriptive statistics were used to summarise sociodemographic, lifestyle, dietary, and sleep-related characteristics of the participants. Normality of continuous variables was assessed using the Shapiro–Wilk test. Results from these tests guided the choice between parametric and non-parametric analyses. Variables following a normal distribution were reported as means and standard deviations (SDs), while non-normally distributed variables were presented as medians and interquartile ranges (IQRs). Categorical variables were expressed as absolute and relative frequencies.

Group comparisons for categorical variables were performed using the chi-square test or Fisher’s exact test, depending not only on the expected cell counts but also on the type of contingency table. Specifically, for larger tables, the chi-square test was used regardless of the cell counts. For continuous variables, normally distributed data were compared using the Student’s *t*-test, whereas non-normally distributed data were analysed using the Mann–Whitney U test (for two-group comparisons) or the Kruskal–Wallis test (for more than two groups). Furthermore, a multiple linear regression model was employed to explore the relationship between adherence to the Mediterranean diet (MEDAS score) and overall sleep quality (global PSQI score), adjusting for potential confounders including sex, age, physical activity, teaching level, and BMI. A two-tailed *p*-value of <0.05 was considered statistically significant for all tests. All statistical analyses were conducted using Jamovi (version 2.4.14; The Jamovi Project, www.jamovi.org; accessed on 23 May 2025).

## 3. Results

### 3.1. General Characteristics

A total of 113 school teachers participated in the study, the majority of whom were female (n = 95; 84%), as shown in Table 1. The median age was 50 (IQR: 45.0–55.0) years. Most participants resided in the Lisbon Metropolitan Area (n = 97; 86%) and held at least a bachelor’s degree (n = 86; 76%). The median length of teaching experience was 23 (IQR: 18.0–30.0) years, with the largest proportion working at the 3rd Cycle education level (n = 27; 38%). Regarding lifestyle and health-related characteristics, the majority were non-smokers (n = 38; 76%) and 44% (n = 22) reported engaging in high levels of physical activity. The median body mass index (BMI) was 23 (IQR: 21.7–26.7) kg/m^2^, with 58% (n = 29) of participants classified as having a normal weight. The total number of responses varied across variables due to missing data.

When stratified by sex, no statistically significant differences were observed. However, there was a trend suggesting that female teachers tended to have more years of professional experience (24.0 vs. 20.0), whereas male teachers exhibited higher BMI values (26.1 vs. 23.1; *p* = 0.065).

### 3.2. Adherence to the Mediterranean Dietary Pattern

Table 2 summarizes participants’ adherence to individual components of the Mediterranean diet, based on responses to the MEDAS questionnaire. Most participants (98%) reported using olive oil as their main culinary fat, although only 32% met the recommended intake of four or more tablespoons per day. More than half consumed more than two servings of vegetables (53%) and at least three pieces of fruit daily (52%). Regarding red or processed meat, 59% of participants reported consuming less than one serving per day. A similar trend was observed for butter, margarine, or cream, with 77% reporting limited intake. Sugar-sweetened or carbonated beverages were consumed by fewer than one unit per day in 91% of the sample. Wine consumption of seven or more glasses per week was reported by 10% of participants. In terms of legumes, 45% reported consuming three or more servings per week. Fish or seafood were consumed more than three times per week by 58% of participants. Intake of commercial baked goods or non-homemade sweets was limited to fewer than three times per week by 72%. Consumption of nuts at a frequency of three or more servings per week was reported by 43%. A preference for lean white meats (chicken, turkey, or rabbit) over red or processed meats was indicated by 76%. In addition, 84% reported eating more than two servings per week of traditional Mediterranean-style dishes prepared with a sautéed base of tomato (or tomato sauce), onion, garlic, and olive oil. The median MEDAS score was 9.0 (IQR: 8.0–10.0).

Concerning sex differences, a significantly higher proportion of men reported consuming fish or seafood compared to women (83% vs. 53%; *p* < 0.05). Although not reaching statistical significance, women tended to report lower intakes of red or processed meats (63% vs. 39%; *p* = 0.55), whereas men showed a tendency toward higher consumption of fruit (72% vs. 48%; *p* = 0.076) and wine (22% vs. 7.4%; *p* = 0.073).

Table 3 presents the general characteristics of participants according to their levels of adherence to the Mediterranean diet. Within the sample, 9% of individuals exhibited low adherence, 57% moderate adherence, and 34% high adherence.

A statistically significant association was found between dietary adherence and the teaching level of the participants. Among primary education teachers, high adherence was most prevalent in primary education 1st Cycle teachers (14/31, 45%), whereas moderate adherence predominated in primary education 3rd Cycle teachers (33/43, 77%). In primary education 2nd Cycle teachers, moderate and high adherence were equally common (7/15, 47% each). Among secondary education teachers, moderate adherence was the most frequently observed (12/24, 50%). No other differences were statistically significant.

### 3.3. Sleep Quality

Table 4 summarises participants’ sleep quality based on the PSQI components. The majority (70%) rated their subjective sleep quality as good. Sleep latency was 16 to 30 min in 41% of participants, and 61% reported a habitual sleep duration of 6 to 7 h per night. Most participants (68%) had sleep efficiency above 85%, and 80% reported minimal weekly sleep disturbances. In addition, 71% indicated not using sleep medication in the previous month. Regarding daytime dysfunction, 64% reported sometimes experiencing difficulties, suggesting a mild impact on daily functioning. The median global PSQI score was 6.0 (IQR 4.0–8.8), and 58% were classified as having poor overall sleep quality. When stratified by adherence to the Mediterranean dietary pattern, no statistically significant differences were observed. For overall sleep quality, the proportion classified as good was 18.2% in the low-adherence group, 43.8% in the moderate-adherence group, and 47.4% in the high-adherence group (Table 4; *p* = 0.215).

Table 5 presents the general characteristics of participants stratified by sleep quality. A statistically significant association was found between sleep quality and teaching level. Among primary education 1st Cycle teachers, poor sleep quality was most prevalent (24/31, 77%). In the 2nd Cycle, good sleep quality was more common (10/15, 67%). For the 3rd Cycle, poor sleep quality predominated (23/43, 53%). Among secondary education teachers, poor sleep quality was again the most frequent (13/24, 54%). Overall, poor sleep quality was more prevalent across most teaching levels, with the exception of the primary education 2nd Cycle teachers, among whom good sleep quality was more frequent.

### 3.4. Adherence to the Mediterranean Diet vs. Sleep Quality

Table 6 presents the results of the linear regression analysis exploring association between adherence to the Mediterranean diet (MEDAS score) and overall sleep quality (global PSQI score). A positive though non-significant association was observed. Considering that higher MEDAS scores reflect greater dietary adherence and higher PSQI scores indicate poorer sleep quality, these findings suggest that, in this sample, greater adherence to the Mediterranean diet was weakly, but not significantly, associated with worse overall sleep quality.

## 4. Discussion

This exploratory study investigated the association between adherence to the Mediterranean diet and self-reported sleep quality in a sample of Portuguese primary and secondary school teachers. Although no statistically significant relationship was found between overall dietary adherence and global sleep quality, several relevant sociodemographic, behavioural, and occupational patterns emerged. These findings contribute to the growing body of evidence on the complex and multidimensional interplay between diet, sleep, and work-related factors, particularly in high-demand professions such as teaching.

### 4.1. Sociodemographic and Lifestyle Profile

The study population was predominantly composed of middle-aged women with substantial teaching experience, reflecting the demographic profile of the Portuguese educational sector [58]. Participants exhibited generally favourable health-related indicators, including high levels of physical activity, low smoking prevalence, and a median BMI within the normal range. These findings are consistent with previous studies showing that individuals with higher education levels tend to adopt healthier behaviours, including more balanced diets and regular physical activity [18,40]. However, teaching is a profession often associated with high levels of psychological stress, administrative burden, and time pressure, which may interfere with health-promoting routines [24,25]. Chronic exposure to such stressors has been linked to emotional exhaustion, sleep disturbances, and unhealthy coping behaviours, including irregular eating patterns and increased consumption of processed foods [26,27,28]. While these behaviours were not predominant in our sample, they remain important considerations when interpreting the results.

### 4.2. Adherence to the Mediterranean Dietary Pattern

Although nearly all participants reported using olive oil as their main culinary fat, fewer than one-third met the recommended intake of four or more tablespoons per day. Intake of fruits, vegetables, legumes, fish, and traditional Mediterranean-style dishes was moderate to high, whereas the consumption of red and processed meats, butter, margarine or cream, sugary beverages, and commercially baked goods was relatively limited. Overall, these patterns reflect a generally favorable dietary profile, though not reaching the threshold of high Mediterranean diet adherence [2,23]. Sex-based differences in dietary behaviours were also observed, although not statistically significant. Women tended to consume more vegetables, olive oil, and home-prepared meals, while men reported higher intake of fish, wine, and sugary or carbonated beverages, as well as greater consumption of fruit and pulses. These findings are consistent with previous studies reporting gendered patterns in food choice, where women are generally more likely to adopt plant-rich and health-oriented diets [29,30].

A significant association was observed between teaching level and adherence to the Mediterranean diet. Among primary education teachers, high adherence was most prevalent in the 1st Cycle, moderate adherence predominated in the 3rd Cycle, and in the 2nd Cycle, moderate and high adherence were equally common. In contrast, among secondary education teachers, moderate adherence was the most frequently reported. These findings suggest a non-uniform distribution across teaching levels, which may reflect differences in workload, teaching responsibilities, and scheduling flexibility. Previous research has highlighted that time scarcity and high occupational demands can hinder meal planning, limit the purchase of fresh ingredients, and reduce opportunities for home cooking, thereby negatively impacting overall dietary quality [28,39].

### 4.3. Sleep Quality

As anticipated, most teachers reported experiencing poor sleep quality, a finding consistent with previous studies in occupational and adult populations [24,25,51,59,60,61,62,63,64]. The profession is both emotionally and cognitively taxing, encompassing long working hours, administrative duties, and the continuous challenge of addressing diverse student needs. Such stressors have been consistently linked to fatigue, burnout, and sleep disturbances [24,25,26,27].

Although PSQI assessments indicated that a substantial proportion of participants experienced poor sleep quality, most reporting subjectively good sleep, highlighting a discrepancy between global PSQI scores and perceived sleep quality. This divergence aligns with prior research showing that self-reported sleep perception may not always correspond to comprehensive sleep quality measures, as individual components of sleep, such as latency, duration, and disturbances, can influence the overall score independently of subjective feeling of restfulness [62,65,66].

Sleep duration and efficiency were within recommended ranges for the majority of the sample, and few participants reported frequent use of sleep medication. Subjective sleep quality, prolonged sleep latency, and the use of medication emerged as the strongest contributors to poor global sleep quality, all of which are well-established determinants of restfulness and recovery [10,23,67]. A statistically significant association was identified between teaching level and sleep quality, with poor sleep quality being more prevalent across most teaching levels, except among primary education 2nd Cycle teachers, for whom good sleep quality was more frequent. Consistent with these observations, Gluschkoff, et al. [68] demonstrated that primary school teachers working in stressful psychosocial environments not only experience elevated work-related stress but also display increased symptoms of poor mental health and sleep deprivation compared to other professional groups. Similarly, Yang, et al. [69] reported that individuals working in primary schools exhibited a higher prevalence of sleep disturbances.

Although sex differences in sleep quality did not reach statistical significance, female participants tended to report poorer sleep, a trend also observed in previous studies examining adult and occupational populations. The gender differences observed in sleep quality among teachers may be explained by a combination of biological, physiological, and psychosocial factors. Women’s sleep patterns are influenced by significant differences in the concentrations of sleep-regulating hormones and the fluctuations of these hormones across the menstrual cycle and life stages, particularly involving ovarian steroids [70]. In addition, affective disorders and socioeconomic disparities, which disproportionately affect women, may act as mediating factors contributing to poorer sleep quality in female populations [71]. Biological and psychosocial factors, as well as differences in help-seeking behaviours and sleep hygiene practices, may underlie these patterns [12,31,72].

### 4.4. Adherence to the Mediterranean Diet vs. Sleep Quality

Examination of global PSQI scores showed no statistically significant differences across Mediterranean diet adherence groups. As shown in Table 4, the proportion of participants with good overall sleep was 18.2% in the low-adherence group, 43.8% in the moderate-adherence group, and 47.4% in the high-adherence group; these differences were not statistically significant.

Analysis of PSQI component scores did not reveal statistically significant differences across adherence groups. Distributions were broadly similar, including for sleep latency. For example, the share with latency under 30 min was comparable between groups (≤15 min: 54.5% low, 31.3% moderate, 28.9% high; 16–30 min: 18.2%, 40.6%, 47.4%; total under 30 min: 72.7%, 71.9%, 76.3%).

Although biological mechanisms could plausibly link Mediterranean diet patterns with sleep regulation, our cross-sectional design and reliance on self-reported MEDAS and PSQI limit causal inference. Nutrients such as tryptophan, magnesium, B vitamins, and omega-3 fatty acids may support neurotransmitter synthesis and circadian regulation [6,19,20,73]. Polyphenols abundant in plant-based foods and olive oil exhibit anti-inflammatory and antioxidant activity that may reduce systemic inflammation and oxidative stress, both linked to sleep disturbances [21,22,74,75]. Nevertheless, the cross-sectional design and reliance on self-reported tools like MEDAS and PSQI limit the ability to detect such effects [2,18,23,67]. A bidirectional relationship between diet and sleep has also been proposed, whereby poor sleep can increase cravings for energy-dense foods and reduce self-regulation, while inadequate diet quality may impair sleep via metabolic dysregulation and inflammation [31,32,33,34]. These mechanistic considerations are therefore speculative and secondary to the null association observed here.

In multivariable linear regression, adherence to the Mediterranean diet was not associated with global PSQI. The point estimate suggested a weak positive direction of effect (higher MEDAS associated with higher PSQI), but this was not statistically significant. Sampling variability, unmeasured confounding factors such as occupational stress, chronotype or mental health, and measurement limitations inherent to self-reported tools like the MEDAS and PSQI may explain this pattern [13,76,77,78,79].

In particular, as shown in the original validation study by Buysse, Reynolds, Monk, Berman and Kupfer [56], and confirmed in more recent reviews [77], the PSQI scoring approach can classify individuals who are effectively managing sleep issues with medication as having poorer sleep quality, even if they report satisfactory rest.

### 4.5. Strengths and Limitations

This study provides novel insights into the relationship between adherence to the Mediterranean diet and sleep quality in an underrepresented professional group. Its strengths include the use of validated instruments (MEDAS and PSQI), detailed sociodemographic and lifestyle characterisation of participants, and the focus on an occupational population exposed to known health risks. The stratification by teaching level and the inclusion of physical activity, smoking status, and BMI further contribute to the robustness of the findings. Nonetheless, several limitations must be acknowledged. First, the cross-sectional design limits the ability to infer causality. The direction of the relationship between diet and sleep remains unclear, particularly given the possibility of bidirectional influences. Second, the relatively small sample size (n = 113), although adequate for exploratory purposes, may have limited the statistical power to detect subtle associations or interaction effects, particularly after stratification by teaching level or other variables. Therefore, the lack of statistical significance in some comparisons should be interpreted with caution. Third, all variables were self-reported, which may introduce recall or social desirability bias. This is particularly relevant for dietary intake and sleep behaviours, which are prone to under- or overestimation. The MEDAS questionnaire, although validated, does not capture total energy intake, meal timing, or portion sizes, all of which may influence sleep quality. Similarly, the PSQI assesses perceived sleep quality but does not include objective measures such as actigraphy or polysomnography. Fourth, potential confounders such as occupational stress levels, mental health status, chronotype, or specific nutrient biomarkers were not included. These factors could play a significant role in mediating the relationship between diet and sleep, especially in high-stress occupations such as teaching [13,27,34]. Finally, the findings of this study should be interpreted with caution regarding their generalisability. The sample consisted exclusively of Portuguese primary and secondary school teachers, which may limit the applicability of the results to other professional groups, cultural contexts, or geographic regions. Differences in educational systems, work conditions, and lifestyle factors could influence the relationship between dietary patterns and sleep quality, and therefore replication in diverse populations is warranted.

Given the absence of statistically significant associations in our data, any mechanistic considerations are presented as hypothesis-generating and should not be construed as evidence of effect. Nutrients such as tryptophan, magnesium, B vitamins, and omega-3 fatty acids may support neurotransmitter synthesis and circadian regulation, and polyphenols abundant in plant-based foods and olive oil may exert anti-inflammatory and antioxidant effects relevant to sleep physiology [6,19,20,73,74,75]. A bidirectional relationship between diet and sleep has also been proposed, whereby poor sleep may increase cravings for energy-dense foods and reduce self-regulation, while inadequate diet quality may impair sleep via metabolic dysregulation and inflammation [31,32,33,34]. These notes should be read in light of this study’s design, sample size, use of self-reported tools, and unmeasured confounding, which constrain causal inference and may obscure small effects.

Future studies should consider employing mixed-method or longitudinal designs, incorporate objective measurements of sleep and dietary intake, and explore mediating variables such as stress, circadian preference, or inflammatory markers. Such approaches would offer a more comprehensive understanding of how the Mediterranean dietary pattern may influence sleep quality in occupational settings.

## 5. Conclusions

In this sample of Portuguese school teachers, we found no statistically significant association between adherence to the Mediterranean diet and global sleep quality. Poor sleep was common overall (median PSQI 6.0, IQR 4.0–8.8; 58% classified as poor sleepers), with non-uniform patterns across teaching levels: poor sleep predominated in most levels except the 2nd Cycle, and moderate adherence to the Mediterranean diet was the most frequent category in several groups. These patterns are descriptive and should not be interpreted causally. Given the cross-sectional design, reliance on self-reported measures, modest sample size after stratification, and potential unmeasured confounding, any inferences beyond these observations are limited. Future research using larger, longitudinal designs and objective assessments of diet and sleep, while accounting for stress, chronotype and other relevant factors, is warranted to clarify whether and how Mediterranean-style eating relates to sleep health in occupational settings.

## Figures and Tables

**Table 1:** Sociodemographic, professional, and lifestyle characteristics of the participants stratified by sex.

	All Population (n = 113)	Male (n = 18)	Female (n = 95)	*p*-Value
Age, median (IQR)	50.0 (45.0–55.0)	51.0 (46.0–55.0)	49.0 (44.5–56.0)	0.972 ^a^
Residence Area, % (n)				
Metropolitan Area of Lisbon	85.8 (97)	83.3 (15)	86.3 (82)	0.583 ^b^
Centro Region	11.5 (13)	16.7 (3)	10.5 (10)	
Autonomous Region of Madeira	2.7 (3)	0.0 (0)	3.2 (3)	
Educational Level Attended, % (n)				
Bachelor’s Degree	76.1 (86)	61.1 (11)	78.9 (75)	0.104 ^b^
Master’s/Doctorate	23.9 (27)	38.9 (7)	21.1 (20)	
Years of Teaching Experience, median (IQR)	23.0 (18.0–30.0)	20.0 (15.2–26.5)	24.0 (20.0–32.0)	0.065 ^a^
Teaching Level, %				
Primary Education 1st Cycle	27.4 (31)	27.8 (5)	27.4 (26)	0.543 ^b^
Primary Education 2nd Cycle	13.3 (15)	11.1 (2)	13.7 (13)	
Primary Education 3rd Cycle	38.1 (43)	27.8 (5)	40.0 (38)	
Secondary Education	21.2 (24)	33.3 (6)	18.9 (18)	
Body Mass Index, median (IQR) (n = 50)	23.1 (21.7–26.7)	26.1 (23.4–28.7)	23.1 (21.3–26.6)	0.065 ^a^
Nutritional Status, % (n) (n = 50)				
Underweight	2.0 (1)	0.0 (0)	2.4 (1)	0.333 ^b^
Normal weight	58.0 (29)	33.3 (3)	63.4 (26)	
Overweight	32.0 (16)	55.6 (5)	26.8 (11)	
Obesity	8.0 (4)	11.1 (1)	7.3 (3)	
Smoking habits, % (n) (n = 50)				
Non-Smoker	76.0 (38)	66.7 (6)	78.1 (32)	0.668 ^c^
Smoker	24.0 (12)	33.3 (3)	22.0 (9)	
Levels of physical activity, % (n) (n = 50)				
Low	18.0 (9)	0.0 (0)	22.0 (9)	
Moderate	38.0 (19)	33.3 (3)	39.0 (16)	0.190 ^b^
High	44.0 (22)	66.7 (6)	39.0 (16)	

Data are presented as percentages (absolute values) or median (interquartile range) for categorical or continuous variables, respectively. Group comparisons were assessed using ^a^ Mann–Whitney U test, ^b^ Chi-squared test or ^c^ Fisher’s exact test, as appropriate. Shapiro–Wilk tests indicated non-normal distribution for all continuous variables (*p* < 0.05); therefore, non-parametric tests were used in subsequent analyses. Abbreviations: IQR: interquartile range.

**Table 2:** Mediterranean Diet Adherence Screener (MEDAS) components stratified by sex.

	Criteria for 1 Point	All Population (n = 113)	Male (n = 18)	Female (n = 95)	*p*-Value
1. Do you use olive oil as your main culinary fat?	Yes	98.2 (111)	94.4 (17)	98.9 (94)	0.294 ^a^
2. How much olive oil do you consume per day (including for frying, seasoning, salad dressing, meals eaten outside the home, etc.)?	≥4 Tablespoons	31.9 (36)	22.2 (4)	33.7 (32)	0.417 ^a^
3. How many servings of vegetables do you consume per day?	≥2	53.1 (60)	44.4 (8)	54.7 (52)	0.422 ^b^
4. How many pieces of fruit (including natural fruit juices) do you consume per day?	≥3	52.2 (59)	72.2 (13)	48.4 (46)	0.076 ^a^
5. How many servings of red meat, hamburgers, or meat products (ham, sausage, etc.) do you consume per day?	<1	59.3 (67)	38.9 (7)	63.2 (60)	0.055 ^b^
6. How many servings of butter, margarine, or cream do you consume per day?	<1	77.0 (87)	72.2 (13)	77.9 (74)	0.600 ^b^
7. How many sugary or carbonated beverages do you drink per day?	<1	91.2 (103)	94.4 (17)	90.5 (86)	1.000 ^a^
8. How many glasses of wine do you drink per week?	≥7 glasses	9.7 (11)	22.2 (4)	7.4 (7)	0.073 ^a^
9. How many servings of pulses do you consume per week?	≥3	45.1 (51)	61.1 (11)	42.1 (40)	0.137 ^b^
10. How many servings of fish or seafood do you consume per week?	≥3	57.5 (65)	83.3 (15)	52.6 (50)	0.019 ^a^
11. How many times do you consume commercial bakery products or sweets (non-homemade), such as cakes, cookies, biscuits per week?	<3	71.7 (81)	72.2 (13)	71.6 (68)	0.956 ^b^
12. How many servings of nuts (walnuts, almonds, including peanuts) do you consume per week?)	≥3	42.5 (48)	33.3 (6)	44.2 (42)	0.392 ^b^
13. Do you preferentially consume chicken, turkey, or rabbit instead of beef, pork, hamburger, or sausage?	Yes	76.1 (86)	77.8 (14)	75.8 (72)	1.000 ^b^
14. How many times per week do you consume boiled vegetables, pasta, rice, or other dishes cooked with a sautéed base (tomato, onion, leek or garlic, and olive oil)?	≥2	84.1 (95)	72.2 (13)	86.3 (82)	0.134 ^b^
MEDAS score		9.0 (8.0–10.0)	9.50 (7.3–10.0)	9.0 (7.0–10.0)	0.671 ^c^

Data are presented as percentages (absolute values). Group comparisons were assessed using ^a^ Fisher’s exact test, ^b^ Chi-squared test or ^c^ Mann–Whitney test, as appropriate.

**Table 3:** Sociodemographic, professional, and lifestyle characteristics stratified by Mediterranean dietary pattern adherence.

	Low Adherence (n = 11)	Moderate Adherence (n = 64)	High Adherence (n = 38)	*p*-Value
Age, median (IQR)	49.0 (46.0–53.50)	48.50 (43.8–55.0)	52.50 (46.0–55.8)	0.472 ^a^
Sex, % (n)				
Male	27.27 (3)	9.4 (6)	23.7 (9)	0.090 ^b^
Female	72.73 (8)	90.6 (58)	76.3 (29)	
Residence Area, % (n)				
Metropolitan Area of Lisbon	63.6 (7)	89.1 (57)	86.8 (33)	0.251 ^b^
Centro Region	27.27 (3)	9.4 (6)	10.4 (4)	
Autonomous Region of Madeira	9.1 (1)	1.6 (1)	2.6 (1)	
Educational Level Attended, % (n)				
Bachelor’s Degree	72.7 (8)	75.0 (48)	78.9 (30)	0.869 ^b^
Master/Doctorate	27.3 (3)	25.0 (16)	21.1 (8)	
Years of Teaching Experience, median (IQR)	23.0 (19.5–28.0)	24.0 (19.5–32.0)	22.0 (18.3–30.0)	0.864 ^a^
Teaching Level, % (n)				
Primary Education 1st Cycle	45.5 (5)	18.8 (12)	36.8 (14)	0.043 ^b^
Primary Education 2nd Cycle	9.1 (1)	10.9 (7)	18.4 (7)	
Primary Education 3rd Cycle	18.2 (2)	51.6 (33)	21.1 (8)	
Secondary Education	27.3 (3)	18.8 (12)	23.7 (9)	
Body Mass Index, median (IQR) (n = 50)	23.0 (21.1–26.8)	23.1 (22.9–23.1)	23.9 (21.8–27.3)	0.682 ^a^
Nutritional Status, % (n) (n = 50)				
Underweight	0.0 (0)	0.0 (0)	6.3 (1)	0.739 ^b^
Normal weight	80.0 (4)	55.2 (16)	56.3 (9)	
Overweight	20.0 (1)	34.5 (10)	31.2 (5)	
Obesity	0.0 (0)	10.3 (3)	6.3 (1)	
Smoking habits, % (n) (n = 50)				
Non-Smoker	80.0 (4)	72.4 (21)	81.3 (13)	0.783 ^b^
Smoker	20.0 (1)	27.6 (8)	18.8 (3)	
Levels physical activity, % (n) (n = 50)				
Low	20.0 (1)	24.1 (7)	6.3 (1)	0.180 ^b^
Moderate	40.0 (2)	44.8 (13)	25.0 (4)	
High	40.0 (2)	31.0 (9)	68.8 (11)	

Data are presented as percentages (absolute values) or median (interquartile range) for categorical or continuous variables, respectively. Group comparisons were assessed using ^a^ Kruskal–Wallis test or ^b^ Chi-squared test, as appropriate. Abbreviations: IQR: interquartile range.

**Table 4:** Pittsburgh Sleep Quality Index components stratified by Mediterranean diet adherence.

	All Population (n = 113)	Low Adherence (n = 11)	Moderate Adherence (n = 64)	High Adherence (n = 38)	*p*-Value
Subjective Sleep Quality					
Very good	5.3 (6)	0.0 (0)	6.3 (4)	5.3 (2)	0.742 ^a^
Good	65.5 (74)	54.5 (6)	35.9 (42)	68.4 (26)	
Poor	25.7 (29)	45.5 (5)	23.4 (15)	23.7 (9)	
Very poor	3.5 (4)	0.0 (0)	4.7 (3)	2.6 (1)	
Sleep Latency					
≤15 min	32.7 (37)	54.5 (6)	31.3 (20)	28.9 (11)	0.682 ^a^
16 to 30 min	40.7 (46)	18.2 (2)	40.6 (26)	47.4 (18)	
31 to 60 min	20.7 (23)	18.2 (2)	21.9 (14)	18.4 (7)	
>60 min	6.2 (7)	9.1 (1)	6.3 (4)	5.3 (2)	
Sleep Duration					
>7 h	28.3 (32)	18.2 (2)	28.7 (19)	28.9 (11)	0.455 ^a^
6 to 7 h	61.1 (69)	54.5 (6)	59.4 (38)	65.8 (25)	
5 to 6 h	9.7 (11)	27.3 (3)	9.4 (6)	5.3 (2)	
<5 h	0.9 (1)	0.0 (0)	1.6 (1)	0.0 (0)	
Habitual Sleep Efficiency					
>85%	68.1 (77)	45.5 (5)	70.3 (45)	71.1 (27)	0.422 ^a^
75% to 84%	21.2 (24)	36.4 (4)	17.2 (11)	23.7 (9)	
65% to 74%	8.0 (9)	9.1 (1)	9.4 (6)	5.3 (2)	
<65%	2.7 (3)	9.1 (1)	3.1 (2)	0.0 (0)	
Sleep Disturbances					
Never	4.4 (5)	0.0 (0)	4.7 (3)	5.3 (2)	0.937 ^a^
Less than once a week	79.6 (90)	90.9 (10)	78.1 (50)	78.9 (30)	
1–2 times/week	15.0 (17)	9.1 (1)	15.6 (10)	15.8 (6)	
≥3 times/week	0.9 (1)	0.0 (0)	1.6 (1)	0.0 (0)	
Sleep Medication					
Never	70.8 (80)	81.8 (9)	73.4 (47)	63.2 (24)	0.818 ^a^
Less than once a week	7.1 (8)	9.1 (1)	6.3 (4)	7.9 (3)	
Once or twice a week	8.8 (10)	0.0 (0)	7.8 (5)	13.2 (5)	
Three or more times per week	13.3 (15)	9.1 (1)	12.5 (8)	15.8 (6)	
Daytime Dysfunction					
Never	15.9 (18)	9.1 (1)	14.1 (9)	21.1 (8)	0.632 ^a^
Sometimes	63.7 (72)	81.8 (9)	60.9 (39)	63.2 (24)	
Often	18.6 (21)	9.1 (1)	21. 9 (14)	15.8 (6)	
Very often	1.8 (2)	0.0 (0)	3.1 (2)	0.0 (0)	
PSQI score, median (IQR)	6.0 (4.0–8.8)	7.0 (6.0–7.5)	6.0 (4.0–8.0)	6.0 (4.0–8.0)	0.643 ^b^
Overall sleep quality					
Poor	57.5 (65)	81.8 (9)	56.3 (36)	52.6 (20)	0.215 ^a^
Good	42.5 (48)	18.2 (2)	43.8 (28)	47.4 (18)	

Data are presented as percentages (absolute values). Group comparisons were assessed using ^a^ Chi-squared test or ^b^ Kruskal–Wallis as appropriate. Abbreviations: IQR: interquartile range.

**Table 5:** Sociodemographic, professional, and lifestyle characteristics stratified by sleep quality.

	Good Sleep Quality (n = 48)	Poor Sleep Quality (n = 65)	*p*-Value
Age, median (IQR)	49.0 (45.0–55.0)	50.0 (44.0–56.0)	0.745 ^a^
Sex, % (n)			
Male	18.8 (9)	13.9 (9)	0.481 ^b^
Female	81.3 (39)	86.2 (56)	
Residence Area, % (n)			
Metropolitan Area of Lisbon	85.4 (41)	86.2 (56)	0.668 ^b^
Centro Region	10.4 (5)	12.3 (8)	
Autonomous Region of Madeira	4.2 (2)	1.5 (1)	
Educational Level Attended, % (n)			
Bachelor’s Degree	75.0 (36)	76.9 (50)	0.813 ^b^
Master/Doctorate	25.0 (12)	23.1 (15)	
Years of Teaching Experience, mean (SD)	21.4 (10.7)	24.7 (9.1)	0.078 ^c^
Teaching Level, %			
Primary Education 1st Cycle	14.6 (7)	36.9 (24)	0.029 ^b^
Primary Education 2nd Cycle	20.8 (10)	7.7 (5)	
Primary Education 3rd Cycle	41.7 (20)	35.4 (23)	
Secondary Education	22.9 (11)	20.0 (13)	
Body Mass Index, mean (SD) (n = 50)	24.9 (4.1)	24.1 (3.8)	0.497 ^c^
Nutritional Status, % (n) (n = 50)			
Underweight	0.0 (0)	3.3 (1)	0.356 ^b^
Normal weight	60.0 (12)	56.7 (17)	
Overweight	25.0 (5)	36.7 (11)	
Obesity	15.0 (3)	3.3 (1)	
Smoking habits, % (n) (n = 50)			
Non-Smoker	80.0 (16)	73.3 (22)	0.740 ^d^
Smoker	20.0 (4)	26.7 (8)	
Levels physical activity, % (n = 50)			
Low	20.0 (4)	16.7 (5)	
Moderate	40.0 (8)	36.7 (11)	0.891 ^b^
High	40.0 (8)	46.7 (14)	
MEDAS score, median (IQR)	9.0 (8.0–10.0)	9.0 (7.0–10.0)	0.287 ^a^

Data are presented as percentages (absolute values) or median (interquartile range) for categorical or continuous variables, respectively. Group comparisons were assessed using ^a^ Mann–Whitney U Test, ^b^ Chi-squared test, ^c^ Student’st test, or ^d^ Fisher’s exact test, as appropriate. Abbreviations: IQR: interquartile range; SD: standard deviation.

**Table 6:** Association between Mediterranean diet adherence and global sleep quality.

	Unstandardized Coefficient	Standard Error	Standardized Coefficient	95% Confidence Interval	*p*-Value
MEDAS score	0.245	0.316	0.124	−0.200 to 0.449	0.443

## Data Availability

Data are contained within the article.

## Supplementary Materials

The following supporting information can be downloaded at: https://www.mdpi.com/article/10.3390/nu17182948/s1, Table S1. STROBE Statement—Checklist of items that should be included in reports of cross-sectional studies.
